# Preoperative Gadoxetic Acid-Enhanced MRI and Simultaneous Treatment of Early Hepatocellular Carcinoma Prolonged Recurrence-Free Survival of Progressed Hepatocellular Carcinoma Patients after Hepatic Resection

**DOI:** 10.1155/2014/641685

**Published:** 2014-02-19

**Authors:** Masanori Matsuda, Tomoaki Ichikawa, Hidetake Amemiya, Akira Maki, Mitsuaki Watanabe, Hiromichi Kawaida, Hiroshi Kono, Katsuhiro Sano, Utaroh Motosugi, Hideki Fujii

**Affiliations:** ^1^First Department of Surgery, Yamanashi University School of Medicine, 1110 Shimokato, Chuo City, Yamanashi 409-3898, Japan; ^2^Department of Radiology, Yamanashi University School of Medicine, 1110 Shimokato, Chuo City, Yamanashi 409-3898, Japan

## Abstract

*Background/Purpose*. The purpose of this study was to clarify whether preoperative gadoxetic acid-enhanced magnetic resonance imaging (EOB-MRI) and simultaneous treatment of suspected early hepatocellular carcinoma (eHCC) at the time of resection for progressed HCC affected patient prognosis following hepatic resection. *Methods*. A total of 147 consecutive patients who underwent their first curative hepatic resection for progressed HCC were enrolled. Of these, 77 patients underwent EOB-MRI (EOB-MRI (+)) before hepatic resection and the remaining 70 patients did not (EOB-MRI (−)). Suspected eHCCs detected by preoperative imaging were resected or ablated at the time of resection for progressed HCC. *Results*. The number of patients who underwent treatment for eHCCs was significantly higher in the EOB-MRI (+) than in the EOB-MRI (−) (17 versus 6; *P* = 0.04). Recurrence-free survival (1-, 3-, and 5-year; 81.4, 62.6, 48.7% versus 82.1, 41.5, 25.5%, resp., *P* < 0.01), but not overall survival (1-, 3-, and 5-year; 98.7, 90.7, 80.8% versus 97.0, 86.3, 72.4%, resp., *P* = 0.38), was significantly better in the EOB-MRI (+). Univariate and multivariate analyses showed that preoperative EOB-MRI was one of the independent factors significantly correlated with better recurrence-free survival. *Conclusions*. Preoperative EOB-MRI and simultaneous treatment of eHCC prolonged recurrence-free survival after hepatic resection.

## 1. Introduction

Hepatocellular carcinoma (HCC) is one of the most malignant tumors worldwide. Hepatic resection is still the most effective treatment for HCC; however, the recurrence rate is very high even after curative resection. The postoperative 5-year recurrence rate was shown to be higher than 70%, with 80% to 95% of recurrence being confined to the liver [1–4]. Intrahepatic recurrence has been classified as either metachronous multicentric-occurrence HCC (MC) or intrahepatic metastasis (IM) [5, 6]. Anatomic hepatic resection has been shown to be effective for micro IM within resected sections or segments with progressed HCC [7–9] but is ineffective for MC in the remnant liver. Recent studies have shown that hypovascular early HCC (eHCC), which is not an indication for resection, progresses to conventional hypervascular HCC. Hypovascular eHCC is thought to be one of the causes of multicentric recurrence of hypervascular HCC after hepatic resection. However, the effects of simultaneous treatment of suspected eHCC at the time of hepatic resection for progressed HCC on postoperative recurrence have never been evaluated.

A new magnetic resonance imaging (MRI) contrast medium, gadoxetic acid, or gadolinium ethoxybenzyl diethylenetriamine pentaacetic acid (Gd-EOB-DTPA) (Primovist, Bayer Healthcare, Osaka, Japan), which has the properties of both an extracellular gadolinium chelate and liver-specific (hepatocyte-targeting) contrast material, has become available [10–13]. Our previous study revealed that Gd-EOB-DTPA-enhanced MRI (EOB-MRI) was the most useful imaging technique for evaluating small HCC, including eHCC [13].

The purpose of this study was to clarify whether preoperative EOB-MRI and simultaneous treatment of suspected eHCC at the time of resection for progressed HCC affected the overall and recurrence-free survival of patients after initial hepatic resection for HCC.

## 2. Materials and Methods

### 2.1. Patients

A total of 147 consecutive progressed HCC patients without extrahepatic metastasis who underwent their first curative hepatic resection for HCC at the First Department of Surgery (Yamanashi University Hospital, Yamanashi, Japan) between 1 January 2005 and 31 December 2010 were retrospectively enrolled in this study. No postoperative death or in-hospital death was reported among these patients.

This study protocol followed the ethical guidelines of the Declaration of Helsinki amended in 2008, and written informed consent was obtained from each patient.

### 2.2. Preoperative Imaging Diagnosis

Preoperative imaging studies, including chest radiography, abdominal ultrasonography (AUS), computed tomography (CT), MRI, hepatic arteriography, CT during arterial portography (CTAP), and CT during hepatic arteriography (CTHA), were performed. One patient did not undergo contrast-enhanced CT, CTHA, or CTAP because of an allergy to the iodinated contrast material. In addition, CTHA and CTAP were not performed in one patient with mild renal dysfunction. EOB-MRI was introduced into our institute in January 2008. Since then, EOB-MRI has been performed before hepatic resection for HCC as essential imaging diagnostics. In this study, 77 patients underwent EOB-MRI (EOB-MRI (+) group) and the remaining 70 patients underwent conventional MRI (EOB-MRI (−) group) before hepatic resection for HCC. Hypovascular hepatic nodules showing low attenuation on unenhanced CT and CTAP and those showing low signal intensity on hepatocyte-phase images of EOB-MRI were diagnosed as suspected eHCCs [13, 14].

### 2.3. Indication of Hepatic Resection

Liver function reserve was assessed by liver biochemistry, Child-Pugh grading [15], and the indocyanine green retention rate at 15 min (ICGR15). Only patients with Child-Pugh class A and ICGR15 below 20% were offered major hepatic resection, which is defined as resection of two or more segments of the liver according to Couinaud's classification [16]. Patients with Child-Pugh class A and ICGR15 more than 20% and selected class B patients underwent minor hepatic resection, which is defined as resection of one segment or less of the liver. Suspected eHCCs detected by preoperative imaging diagnosis with or without EOB-MRI, and being more than 5 mm in diameter, were simultaneously resected or ablated at the time of resection for progressed HCC.

### 2.4. Pathological Diagnosis

All surgically resected specimens were fixed in 10% buffered formaldehyde. Sections of resected tumors and noncancerous livers were embedded in paraffin, sliced into 3 to 5 *μ*m thick sections, and were then stained with hematoxylin-eosin for histological analyses. All suspected eHCCs were diagnosed strictly according to the pathological criteria proposed by the International Consensus Group for Hepatocellular Neoplasia (ICGHN) [17] by pathologists.

### 2.5. Postoperative Follow-Up

After the initial operation, patients were followed up at 2-week intervals for the first 2 months and monthly thereafter. Serum levels of *α*-fetoprotein (AFP), a *Lens culinaris* agglutinin reactive fraction of AFP (AFP-L3), and des-*γ*-carboxy prothrombin (DCP) were measured serially at least every 2 months. Imaging diagnosis, using CT or MRI, was performed at least every 4 months. When intrahepatic recurrence was suspected, the patient was hospitalized for diagnosis and treatment. Diagnosis of recurrence was made when intrahepatic hypervascular HCC was found by contrast-enhanced CT, MRI, or CTHA.

### 2.6. Treatment Strategy for Intrahepatic Recurrence of HCC

If intrahepatic recurrence was ≤3 nodules and all tumors were potentially resectable in terms of anatomical location and liver function, recurrence was managed using repeat hepatic resection. Recurrence was managed using radiofrequency ablation (RFA) if intrahepatic recurrence was solitary and completely ablative and hepatic function of the patient was not suitable for repeat hepatic resection or if the patient refused hepatic resection. Multiple intrahepatic recurrence (>3 nodules) was treated using transcatheter arterial chemoembolization (TACE) [18].

### 2.7. Statistical Analysis

Continuous data are expressed as the median (range) and were compared using the Mann-Whitney *U* test. Categorical variables were compared using the Chi-square test with Yates' correction or Fisher's exact test where appropriate. Survival was calculated by the Kaplan-Meier method and compared by means of the log-rank test. Univariate and multivariate analyses were performed using the Cox proportional hazards model to identify prognostic factors. Only differences with probability values below 0.05 were considered significant.

## 3. Results

### 3.1. Comparison of Background Factors between the Two Groups

A comparison of background factors between the EOB-MRI (+) and EOB-MRI (−) groups was performed (Table 1). The DCP value of patients in the EOB-MRI (−) group was significantly higher than that in the EOB-MRI (+) group (median: 57 mAU/mL; range: 12–30805 mAU/mL; versus 29.0 mAU/mL; 9–17521 mAU/mL; *P* = 0.02). The number of patients who underwent treatment for eHCCs at the time of hepatic resection was significantly higher in the EOB-MRI (+) group than in the EOB-MRI (−) group (17 versus 6; *P* = 0.04). No significant differences were observed in the other background factors between groups.

### 3.2. Simultaneous Treatment of Suspected eHCCs at the Time of Resection and Histological Evaluation of the Tumors

In the EOB-MRI (−) group, 8 suspected eHCCs from 6 patients were resected at the time of the operation. Histologically, 6 out of 8 resected tumors were eHCC and two were dysplastic nodules (DNs). On the other hand, 24 suspected eHCCs from 17 patients were treated in the EOB-MRI (+) group (23 were resected and one was ablated by microwaves without biopsy) at the time of the operation. Histological examination revealed that 21 out of 23 resected tumors were eHCC, one was DN, and one was accessory liver. In the EOB-MRI (+) group, 8 out of 21 eHCCs (38.1%) were detected at only hepatocyte phase of EOB-MRI.

### 3.3. Comparison of Overall and Recurrence-Free Survival after Hepatic Resection between the Two Groups

**Figure 1:**
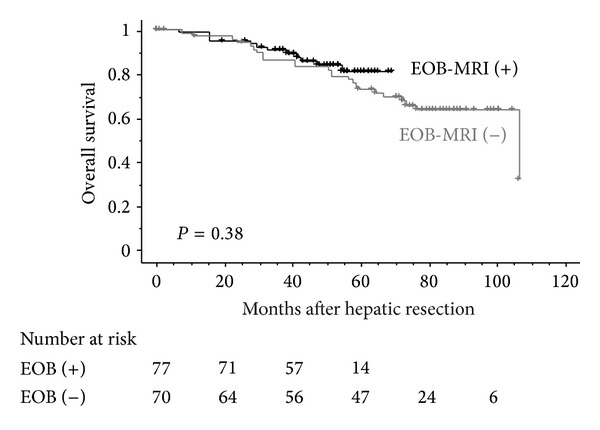
Overall survival curves of patients stratified according to the presence or absence of preoperative EOB-MRI after hepatic resection for progressed HCC.

**Figure 2:**
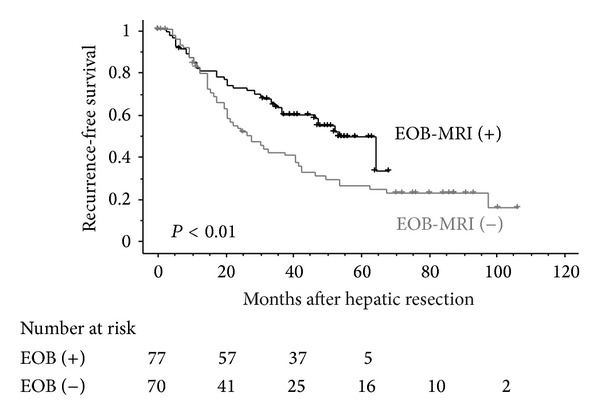
Recurrence-free survival curves of patients stratified according to the presence or absence of preoperative EOB-MRI after hepatic resection for progressed HCC.

The 1-, 3-, and 5-year overall survival rates of 77 HCC patients in the EOB-MRI (+) group were 98.7, 90.7, and 80.8%, respectively, whereas the corresponding survival rates of 70 HCC patients in the EOB-MRI (−) group were 97.0, 86.3, and 72.4%, respectively (Figure 1). No significant differences were observed in the overall survival curves between the two groups (*P* = 0.38). On the other hand, the 1-, 3-, and 5-year recurrence-free survival rates of 77 HCC patients in the EOB-MRI (+) group were 81.4, 62.6, and 48.7%, respectively, whereas those of 70 HCC patients in the EOB-MRI (−) group were 82.1, 41.5, and 25.5%, respectively (Figure 2). Recurrence-free survival after hepatic resection was significantly better in the EOB-MRI (+) group than in the EOB-MRI (−) group (*P* < 0.01).

### 3.4. Univariate Analysis of Prognostic Factors for Overall and Recurrence-Free Survival after Hepatic Resection for HCC

Tables 2 and 3 summarize the results of univariate analysis of the 20 clinical and laboratory factors and 7 pathological and tumor-related factors, respectively, for overall survival and recurrence-free survival among the 147 patients with HCC following curative hepatic resection.

In clinical and laboratory factors, the presence of an esophageal varix (*P* = 0.02), platelet count 10 × 10^4^/*μ*L or less (*P* < 0.01), Child-Pugh class B (*P* < 0.01), and a DCP value more than 40 mAU/mL (*P* = 0.03) were correlated with significantly worse overall survival (Table 2). Other clinical and laboratory factors did not show any significant influence on overall survival after hepatic resection for HCC. In pathological and tumor-related factors, the presence of liver cirrhosis (*P* = 0.01) was correlated with significantly worse overall survival (Table 3).

In clinical and laboratory factors, preoperative EOB-MRI significantly reduced the risk of recurrence (*P* < 0.01). On the other hand, a serum albumin level of 3.5 g/dL or less (*P* < 0.01), platelet count 10 × 10^4^/*μ*L or less (*P* = 0.04), Child-Pugh class B (*P* < 0.01), and being positive for hepatitis C antibodies (*P* < 0.05) were correlated with significantly worse recurrence-free survival (Table 2). In pathological and tumor-related factors, multiple progressed HCCs (*P* < 0.01) were correlated with significantly worse recurrence-free survival by univariate analysis (Table 3).

### 3.5. Multivariate Analysis of Prognostic Factors for Overall and Recurrence-Free Survival after Hepatic Resection for HCC

According to multivariate analysis for factors that could influence overall survival, a platelet count 10 × 10^4^/*μ*L or less (HR 4.41, 95% CI 2.04–9.52, *P* < 0.01) and DCP value more than 40 mAU/mL (HR 3.55, 95% CI 1.72–7.34, *P* < 0.01) were selected as independent predictors of adverse overall survival of patients with HCC after hepatic resection (Table 4). On the other hand, preoperative EOB-MRI (HR 0.56, 95% CI 0.36–0.86, *P* < 0.01) and multiple progressed HCCs (HR 2.10, 95% CI 1.29–3.40, *P* < 0.01) were identified as independent factors significantly correlated with recurrence-free survival after hepatic resection (Table 4).

## 4. Discussion

The findings of this retrospective study showed that preoperative EOB-MRI and simultaneous treatment of eHCC prolonged recurrence-free survival but not overall survival of progressed HCC patients following hepatic resection. Univariate and multivariate analyses showed that preoperative EOB-MRI was one of the independent factors significantly correlated with recurrence-free survival after hepatic resection. To the best of our knowledge, this is the first study to show that simultaneous treatment of eHCC based on the preoperative imaging study including EOB-MRI prolonged recurrence-free survival after hepatic resection for HCC.

A new MR imaging contrast medium, gadoxetic acid, or Gd-EOB-DTPA, which has the properties of both an extracellular gadolinium chelate and liver-specific contrast material, has become available. Injection of a bolus of Gd-EOB-DTPA allows for the assessment of tumor vascularity using arterial phase imaging and enables hepatocyte-phase imaging approximately 20 minutes after its administration, with approximately 50% of the contrast material being taken up by hepatocytes [10–13]. EOB-MRI including a gradient dual-echo sequence and diffusion-weighted imaging has been recommended for the pretherapeutic evaluation of patients with HCC [19]. Our previous study showed that EOB-MRI was the most useful imaging technique for evaluating small HCC, including eHCC [13]. Hypovascular nodules that appear hypointense on hepatocyte-phase EOB-MRI may progress to conventional hypervascular hepatocellular carcinoma [20]. We previously showed that nodules more than 10 mm in diameter and containing fat were at a higher risk of developing hypervascularization [14]. Moreover, a maximum diameter more than 10 mm [21] or 15 mm or greater [22], increased growth rate, hyperintensity on T1-weighted images [23], hyperintensity on T2-weighted images, and a tumor volume doubling time of less than 542 days [24] were reported to be risk factors of hypervascularization in hypovascular nodules that appeared hypointense on hepatocyte-phase EOB-MRI.

We showed that preoperative EOB-MRI and simultaneous treatment of eHCC at the time of resection for progressed HCC prolonged recurrence-free survival after hepatic resection. The recurrence-free survival curves of the two groups overlapped within one year after hepatic resection and after that the incidence of recurrence in the EOB-MRI (+) group became significantly lower than that in the EOB-MRI (−) group. We speculate that recurrence within one year of hepatic resection is mainly due to enlargement of preoperatively undetectable intrahepatic micro IM of resected progressed HCC, while recurrence after one year was MC that progressed from eHCC to hypervascular HCC or was *de novo* hypervascular HCC. We estimate that simultaneous treatment of eHCC at the time of resection for progressed HCC reduced MC by removing eHCC that may have progressed to hypervascular HCC.

One of the reasons why preoperative EOB-MRI and simultaneous treatment of eHCC at the time of resection prolonged recurrence-free survival but not overall survival after hepatic resection for HCC was early diagnosis and prompt treatment of recurrent HCC detected by our postoperative close follow-up.

Reducing postoperative recurrence after hepatic resection of HCC may not only diminish the burden on patients, but also preserve liver function.

## 5. Conclusions

The present study showed for the first time that preoperative EOB-MRI and simultaneous treatment of eHCC prolonged recurrence-free survival of progressed HCC patients following hepatic resection. However, because our data were based on a retrospective study and limited number of patients, further prospective studies are required to fully evaluate the significance of preoperative EOB-MRI and simultaneous treatment of eHCC.

## Figures and Tables

**Table 1:** Comparison of background factors between the EOB-MRI (+) and EOB-MRI (−) groups.

Factor	EOB-MRI (+)	EOB-MRI (−)	*P* value
	(*n* = 77)	(*n* = 70)	
Sex (male/female)	57/20	53/17	0.85
Age (years)*	68 (16–86)	69 (35–85)	0.71
History of blood transfusions (present/absent)	22/55	16/54	0.46
History of schistosomiasis japonica (present/absent)	10/67	12/58	0.50
Alcoholism (present/absent)	36/41	32/38	>0.999
Smoking (present/absent)	51/26	44/26	0.73
Diabetes mellitus (present/absent)	18/59	26/44	0.08
Esophageal varix (present/absent)	17/60	12/58	0.54
Albumin (g/dL)*	4.0 (3.1–4.8)	3.9 (3.2–4.7)	0.23
Total Bilirubin (mg/dL)	0.7 (0.3–1.6)	0.7 (0.2–1.4)	0.91
Alanine aminotransferase (IU/L)*	37 (9–168)	37 (10–286)	0.73
Platelets (×10^4^/*μ*L)*	12.4 (3.2–35.6)	13.7 (5.7–32.8)	0.12
Indocyanine green retention rate at 15 minutes (%)*	15.3 (5.4–30.6)	12.6 (5.5–38.0)	0.35
Prothrombin time (%)*	83.2 (54.6–113.5)	78.7 (61.7–100)	0.13
Child-Pugh score (A/B)	73/4	62/8	0.23
Alpha-fetoprotein (ng/mL)*	10.7 (9–17521)	14.3 (1.7–128900)	0.19
Alpha-fetoprotein L3 (%)*	1.2 (0–80.4)	2.1 (0–77.7)	0.33
Des-*γ*-carboxy prothrombin (mAU/mL)*	29.0 (9–17521)	57.0 (12–30805)	0.02
Hepatitis B surface antigen (positive/negative)	16/61	16/54	0.84
Hepatitis C antibodies (positive/negative)	45/32	41/29	>0.999
Tumor size in greatest dimension (cm)*	2.6 (0.9–10.7)	3.0 (1.0–9.0)	0.14
Number of tumors (solitary/multiple)	62/15	48/22	0.13
Fibrous capsule formation (present/absent)	55/22	58/12	0.12
Vascular invasion (present/absent)	14/63	18/52	0.32
Pathological diagnosis (well or moderate/poor)	61/16	57/13	0.84
Liver cirrhosis (present/absent)	41/36	36/34	0.87
AJCC Stage (I/II or III)	51/26	39/31	0.24
Hepatic resection (major/minor)	26/51	21/49	0.73
Treatment for eHCC	17/60	6/64	0.04

*Median (range).

**Table 2:** Univariate analysis of clinical and laboratory factors for overall and recurrence-free survival after hepatic resection for HCC.

Variable	Number of patients	Overall survival			Recurrence-free survival		
		HR	95% CI	*P* value	HR	95% CI	*P* value
Sex							
Male	110	1			1		
Female	37	0.81	0.37–1.79	0.61	0.66	0.39–1.16	0.13
Age (years)							
≦70	85	1			1		
>70	62	1.01	0.97–1.04	0.78	1.33	0.86–2.03	0.2
EOB-MRI							
Absent	70	1			1		
Present	77	0.72	0.34–1.15	0.38	0.57	0.37–0.87	<0.01
History of blood transfusion							
Absent	109	1			1		
Present	38	1.4	0.69–2.87	0.35	1.18	0.73–1.90	0.48
Alcoholism							
Absent	79	1			1		
Present	68	1.4	0.72–2.72	0.32	1.34	0.88–2.05	0.17
Smoking							
Absent	52	1			1		
Present	95	1.66	0.80–3.45	0.17	1.24	0.79–1.94	0.34
Diabetes mellitus							
Absent	103	1			1		
Present	44	1.5	0.76–2.93	0.24	1.32	0.85–2.07	0.22
Esophageal varix							
Absent	118	1			1		
Present	29	2.39	1.17–4.91	0.02	1.24	0.73–2.11	0.44
Albumin (g/dL)							
≦3.5	19	1			1		
>3.5	128	0.5	0.23–1.10	0.08	0.49	0.28–0.84	<0.01
Total bilirubin (mg/dL)							
≦1.0	133	1			1		
>1.0	14	0.42	0.10–1.78	0.24	0.71	0.34–1.49	0.36
Alanine aminotransferase (IU/L)							
≦30	59	1			1		
>30	88	1.44	0.71–2.95	0.31	1.5	0.96–2.34	0.08
Platelets (×10^4^/*μ*L)							
≦10	33	1			1		
>10	114	0.27	0.27–0.52	<0.01	0.6	0.37–0.97	0.04
Indocyanine green retention rate at 15 minutes (%)							
≦15	77	1			1		
>15	70	1.78	0.90–3.50	0.1	1.13	0.74–1.71	0.58
Prothrombin time (%)							
≦80	71	1			1		
>80	76	0.59	0.30–1.17	0.13	0.71	0.47–1.10	0.11
Child-Pugh classification							
A	137	1			1		
B	10	3.68	1.51–8.93	<0.01	2.65	1.32–5.32	<0.01
Alpha-fetoprotein (ng/mL)							
≦100	117	1			1		
>100	30	0.71	0.29–1.71	0.44	0.71	0.41–1.24	0.23
AFP-L3 (%)							
≦10	110	1			1		
>10	36	0.97	0.46–2.08	0.94	0.79	0.47–1.31	0.36
Des-*γ*-carboxy prothrombin (mAU/mL)							
≦40	76	1			1		
>40	68*	2.14	1.08–4.22	0.03	1.4	0.91–2.14	0.12
Hepatitis B surface antigen							
Negative	115	1			1		
Positive	32	0.97	0.44–2.14	0.94	0.62	0.35–1.10	0.09
Hepatitis C antibody							
Negative	61	1			1		
Positive	86	1.42	0.71–2.86	0.33	1.56	1.01–2.42	<0.05

*Three patients were excluded because of warfarin administration.

**Table 3:** Univariate analysis of pathological and tumor-related factors for overall and recurrence-free survival after hepatic resection for HCC.

Variable	Number of patients	Overall survival			Recurrence-free survival		
		HR	95% CI	*P* value	HR	95% CI	*P* value
Diameter of main tumor (cm)							
≦2.0	39	1			1		
>2.0	108	1.11	0.50–2.46	0.79	1.39	0.84–2.30	0.19
Number of advanced HCCs							
Solitary	110	1			1		
Multiple	37	1.26	0.60–2.62	0.54	1.96	1.22–3.14	<0.01
Fibrous capsule formation							
Absent	34	1			1		
Present	113	1.18	0.51–2.70	0.7	1.26	0.57–2.12	0.39
Vessel invasion							
Absent	115	1			1		
Present	32	0.95	0.43–2.10	0.9	1.03	0.62–1.71	0.91
Pathological diagnosis							
Well or moderate	118	1			1		
Poor	29	1.68	0.78–3.62	0.18	1.02	0.59–1.75	0.95
AJCC stage							
I	90	1			1		
II or III	57	1.29	0.67–2.49	0.45	1.29	0.83–1.98	0.25
Liver cirrhosis							
Absent	70	1			1		
Present	77	2.58	1.23–5.38	0.01	1.23	0.81–1.89	0.34

**Table 4:** Multivariate analysis of prognostic factors for overall and recurrence-free survival after hepatic resection for HCC.

Variable	HR	95% CI	*P* value
Overall survival			
Esophageal varix (present)	1.49	0.65–3.38	0.34
Platelets (≦10 × 10^4^/*μ*L)	4.41	2.04–9.52	<0.01
Child-Pugh classification (B)	1.48	0.53–4.13	0.45
Des-*γ*-carboxy prothrombin (>40 mAU/mL)	3.55	1.72–7.34	<0.01
Liver cirrhosis (present)	1.92	0.89–4.15	0.1
Recurrence-free survival			
EOB-MRI (present)	0.56	0.36–0.86	<0.01
Albumin (≦3.5 g/dL)	1.68	0.87–3.26	0.12
Platelets (≦10 × 10^4^/*μ*L)	1.66	0.99–2.79	0.054
Child-Pugh classification (B)	1.52	0.61–3.79	0.36
Hepatitis C antibody (positive)	1.33	0.94–2.34	0.09
Number of advanced HCCs (multiple)	2.1	1.29–3.40	<0.01
